# Acute post stroke depression at a Primary Stroke Center in the Middle East

**DOI:** 10.1371/journal.pone.0208708

**Published:** 2018-12-20

**Authors:** Stacy Schantz Wilkins, Naveed Akhtar, Abdul Salam, Paula Bourke, Sujatha Joseph, Mark Santos, Ashfaq Shuaib

**Affiliations:** 1 The Neuroscience Institute (Stroke Center of Excellence), Academic Health, Hamad Medical Corporation, Doha, Qatar; 2 VA Greater Los Angeles Healthcare System, Los Angeles, CA, United States of America; 3 Department of Medicine, University of Alberta, Alberta, Canada; Leids Universitair Medisch Centrum, NETHERLANDS

## Abstract

**Objective:**

Depression occurs in approximately 30 percent of stroke patients, leading to increased disability, lower quality of life and increased mortality. Given new recommendations to assess depression in acute stroke patients this study evaluated rates of acute post stroke depression at a Primary Stroke Center in Doha, Qatar.

**Methods:**

Acute stroke patients (n = 233) were given the PHQ-9 and the Mini-Cog test by stroke unit nurses within the first few days post stroke. This was part of a clinical improvement project conducted from March 2016 thru March 2017.

**Results:**

Approximately 20% of acute post stroke patients (46/233) scored in the moderately depressed range on the Patient Health Questionnaire (PHQ-9 ≥10 with item 1 and/or 2 endorsed). Nationality and dysarthria were significantly associated with depression. Females were twice as likely to be depressed. A significantly greater number of Middle Eastern and African patients were depressed (30.18%) than Southeast Asian and Western Pacific patients (16.76%). A PHQ-2 cut off of 2 was optimal with sensitivity of 91.3 and specificity of 71.6.

**Conclusions:**

Almost 20% of acute stroke patients were moderately depressed on the PHQ-9, with Middle Eastern/African patients almost twice as likely to be depressed. This may reflect higher baseline pre-stroke depression levels in those of Middle Eastern/African background, perhaps due to greater levels or stress or trauma exposure in these groups. Dysarthria was found to be significantly associated with depression. Initial screening with the PHQ-2 using a cut-off of 2 (versus the cut-off of 3 used in primary care settings) may be beneficial. Based on these results acute post stroke depression screening is recommended in the Middle East, coupled with culturally sensitive psychiatric care.

## Introduction

Depression is a common consequence of stroke, with meta-analytic studies finding depression in approximately 30 percent of post stroke patients [1]. Post-stroke depression (PSD) rates vary from 20% to 65% depending on the population studied, assessment measures utilized and study definition of depression [1–3]. Screening patients for depression acutely, within the first few days after stroke, has may improve treatment outcomes through earlier intervention [4]. Moderate levels of depression (Patient Health Questionnaire-9 (PHQ-9) ≥10) were found in the United States in 12 percent of acute stroke patients [4].

Patients with PSD have significantly worse outcomes including poorer rehabilitation progress, longer hospital stays and higher rates of morbidity and mortality [1–7]. Treatment of PSD has been found to successfully improve mood, quality of life, rehabilitation outcomes and cognition, and also to increase life span [1,6].

The Patient Health Questionnaire—9 (PHQ-9) is an easy to administer 9 item depression questionnaire that asks patients to rate their symptoms of depression from 0–3, 0 = “not present” to 3 = “present almost every day” [8]. The PHQ-9 is based on the major depression criteria from the Diagnostic and Statistical Manual-5 (DSM-5) [9] and has been validated in medical patients generally and in stroke patients specifically [10]. The PHQ-9 has been translated into multiple languages, and used successfully in many countries around the world including Saudi Arabia [11], India [12], Thailand [13] and Kenya [14].

The PHQ-2 is the short form of the PHQ-9; it consists of the first 2 items from the PHQ-9, depressed mood and loss of interest/pleasure, and yields a score of 0–6, with 3 or greater indicating a positive screen for depression [15]. The PHQ-2 is widely used as a screener for depression in primary care, and in multiple medical settings including post stroke, with patients who score positive receiving the full PHQ-9 [16,10].

Recently, international stroke guidelines have recommended initiating depression screening on acute stroke units by administration of a structured depression inventory such as the Patient Health Questionnaire [17,18].

### Aims

This study aimed to determine base rates of acute post stroke depression in Qatar, including assessment of the sensitivity and specificity of the PHQ-2 on the acute stroke unit at Hamad General Hospital in Doha, Qatar. The PHQ-2 was investigated as an easier to use short form of the PHQ-9 to help improve rates of depression screening.

Secondary aims included the identification of factors associated with depression including demographics (nationality, sex, education), cognitive deficits, cardiovascular risk factors, stroke symptoms and stroke severity.

## Methods

This was a clinical improvement project conducted from March 2016 thru March 2017 on the stroke unit at Hamad General Hospital (HGH). Hamad General Hospital (HGH) is a Joint Commission International (JCI) accredited 600 bed hospital, and serves as the teaching hospital for Weill Cornell Medical College in Qatar. 95% of all strokes in Qatar requiring admission to hospital are admitted to HGH [19].

All patients who were able to communicate and were not confused/delirious were administered the PHQ-9 and the Minicog by the stroke unit nurses as soon as they were stabilized on the acute stroke unit. The PHQ-9 and the Minicog were given by a nurse who spoke the same language as the patient, and the PHQ-9 questions were given in the patient’s first/best language. The PHQ-9 is a depression screening tool discussed in the introduction [8, 20]. Moderate levels of depression on the PHQ-9 are scored when the PHQ-9 ≥10 with item 1 and/or 2 endorsed. The Minicog screens cognitive ability and includes a 3 item recall and drawing a clock with hands set at 10 past 11. The test has high sensitivity and specificity and is widely used as a screening measure of cognition. [20]. A clinical psychologist trained the nursing staff in test administration through didactic sessions and practice administrations of the instrument, and provided follow-up consultation and supervision throughout the project. The psychologist also reviewed all test results twice a week and when patients were available provided a DSM-V based depression mood interview for all patients with PHQ-9 scores of 10 or more in order to provide optimal patient care.

Prior studies of PSD have sometimes used a cutoff of 4 on the PHQ-9, resulting in higher reporting of depression rates. We used the most common, clinically relevant cut off of 10 or greater, moderate range, on the PHQ-9 as it indicates levels of depression in need of treatment intervention [1,8]. Some prior studies of acute stroke have modified the PHQ-9 time duration to be “since your hospitalization” [4]; we gave the standardized instruction for patients to consider how they had felt for the past two weeks including their time since hospitalization.

Demographic and treatment information was collected from the stroke database at HGH. The following data were collected: demographics including age, gender, education and nationality; cardiovascular risk factors including diabetes, hypertension, dyslipidemia, history of prior stroke and current smoking. Stroke symptoms including weakness, speech deficit, posterior circulation symptoms (dizziness, vomiting and reduced LOC), headache, seizure, visual concerns and abnormal behavior. Other variables included National Institutes of Health Stroke Scale (NIHSS), Modified Rankin Scale (mRS) and discharge diagnosis and location.

Of note, the majority of people in Qatar are expatriates and come from other nations to work. Thus, nationality was recorded as a measure of cultural/ethnic background. Given a large number of nationalities, patients were grouped into categories based on similar location.

A power analysis was conducted to determine sample size. A primary objective of the study was to observe prevalence of depression among stroke patients. The prevalence of depression in stroke has been reported to be about 31%^3^. A sample size of 283 subjects was determined to be enough to produce a 95% confidence interval (24.7% to 35.7%) using Binomial distribution when the estimated proportion of depression in stroke patients is 31.0%. 292 patients were screened, 9 had to be removed due to missing data. As data analysis was conducted 50 stroke ward patients were found to be stroke mimics at discharge. They were excluded from the analysis leaving a total sample size of 233.

A multiple binary logistic regression model was used to identify significant independent factors associated with depression after adjusting for potentially confounding factors such as age, and gender. To build the model, a purposeful selection method was used for selecting a subset of covariates considered clinically important, adjusting for confounders and statistical significance. Purposeful selection of covariates began with a multivariate model that contained all variables that were significant in the bivariate analysis at the 20–25 percent level, as well as any other variables not selected with this criterion but judged to be of clinical importance. Variables included in the regression model included Age, Gender, Nationality, NIHSS, HbA1C, DM, HTN, Dyslipidemia, PriorStroke, BMI, Smoking, Dysarthria, LOSindays. We used p-values from the Wald tests of the individual coefficient to identify covariates that might be deleted from the model and p-value of the partial likelihood ratio test confirming that the deleted covariate is not significant. Following the fitting of the reduced model, we assessed whether or not removal of the covariate produced an “important” change (about 20%) in the coefficient of the variables remaining in the model. The final model was assessed by using goodness-of-fit test to see if the model fit the data.

The study was reviewed and approved by the Hamad Medical Corporation Research Internal Review Board, study protocol #16349/16. As this was a retrospective analysis of a clinical improvement project an exemption was approved and individual consent was not needed.

## Results

Demographics and clinical factors in the depressed and non-depressed groups are presented in Table 1. There was a trend for greater hypertension in the depressed group, and females were more depressed. Of note, disability (mRS), stroke severity (NIHSS), prior stroke, Mini-Cog and smoking were not linked with depression. Mean time from stroke until screening was 1.5 days for non-depressed and 1.9 days for depressed, a non-significant difference (p = .13).

**Table 1:** Demographic and clinical factors and PHQ-9 depression.

	PHQ-9 Positive (46)	PHQ-9 Negative (187)	OR (95% CI)	p.Value
**Age**	52.56 ± 10.37	49.81 ± 11.32	1.02 (.99, 1.05)	.13
**Gender** (% by dx group)			0.38 (0.17, 0.86)	.021
Female	11 (35%)	20 (65%)		
Male	35 (17.3%)	167 (82.7%)		
**Nationality (%)**			2.15 (1.06, 4.35)	.034
Arabic (MENA)	16/53 (30.2)	37/53 (69.8)		
Southeast Asian/ Western Pacific	30/179 (16.8)	149/179 (83.2)		
**NIHSS (on arrival)**	4.56 ± 3.84	4.25 ± 3.41	1.03 (.938, 1.12)	.579
**mRS = 0 prior to stroke (%)**	45/46 (97.8)	184/187(98.4)	1.36 (.14, 13.41)	.791
**Hypertension (%)**	36/46 (78)	122/187 (65)	1.92 (.89, 4.10)	.094
**Diabetes (%)**	23/42 (55)	84/159 (53)	1.08 (.55, 2.14)	.823
**Dyslipidemia (%)**	22/46 (48)	87/187 (47)	1.05 (.55, 2.01)	.874
**Prior Stroke (%)**	5/46 (10.86)	11/187 (5.8)	1.95 (.64, 5.92)	.238
**BMI**	27.37 ± 5.69	27.27 ± 4.45	1.00 (.94, 1.07)	.906
**Smoking (%)**	14/46 (30.4)	43/187 (23)	1.46 (.72, 2.99)	.295
**Dysarthria (%)**	19/46 (41.3)	48/187 (25.6)	2.04 (1.04, 3.99)	.038
**MiniCog Pass (%)**	26/45 (58)	121/186 (65)	0.72 (.37, 1.41)	.340
**LOS days**	5.37 ± 3.72	4.52 ± 3.27	1.07 (.98, 1.17)	.131

Approximately twenty percent (46/233) of the acute stroke patients were positive for depression with a PHQ-9 score of 10 or more and either item 1 or 2 endorsed.

Multiple logistic regression identified nationality and dysarthria as significant independently associated with depression (please see Table 2).

**Table 2:** Multiple logistic regression model to identify significantly associated independent variables for depression (n = 233).

	Adjusted Odds Ratio	95% Confidence Interval for AOR	P-value
**Nationality**			
• Arabic (MENA)	2.12	1.04–4.31	0.04
• Southeast Asian & Western Pacific	1		
**Dysarthria (%)**	1.99	1.01–3.93	0.047

P-value has been calculated using binary multiple logistic regression Wald test. The Hosmer-Lemeshow Goodness-of-fit statistics (Chi-Square = 0.73; p-value = 0.69). ARO = adjusted odds ratio.

Table 2 shows only the variables that were statistically significantly associated with depression. Age, gender and other covariates such as HTN, DM were not statistically significant and therefore have been removed from the model. Patients from the Middle East and Africa were more than twice as likely to be depressed than those from Southeast Asia or the Western Pacific; and those who presented with dysarthria were almost twice as likely to be depressed. Type of stroke was not related with depression. The most common stroke types included ischemic subcortical (43.2%), ischemic posterior (21.2%) and ICH (13.7%); only 7.1% were ischemic cortical.

Seventeen of the 46 patients with positive PHQ-9 depression scores received a diagnostic interview by a clinical psychologist. 13/17 (76%) met DSM-5 criteria for a major or minor depression; 8 patients met criteria for a major depressive episode and 5 had significant symptoms of depression or anxiety that would have benefitted from psychotherapy. Of the remaining 4, one appeared to have a comprehension issue, one had primarily pain symptoms and two had acute post stroke stress and anxiety that resolved.

Item analysis was conducted on the PHQ-9 (please see Table 3). The odds ratio in Table 3 were measured to assess the impact of each individual scale of PHQ-9 (PHQ-1 to PHQ-9) on the overall depression score (depressed vs. non-depressed). Odds ratio of 10.8 for PHQ-1 (Anhedonia) indicate that the odds of having depression among patients having score of 2 or more for PHQ-1 is 10.8 times more likely to depressed compare to those who had a PHQ-1 score below 2. The most frequent items endorsed by depressed patients were trouble sleeping, feeling tired, appetite changes and anhedonia (loss of interest or pleasure). The most common symptom in the non-depressed group was also feeling tired, and 10% or more endorsed depressed mood or anhedonia.

**Table 3:** Frequency (%) of stroke patients endorsing each PHQ-9 symptom more than half the days or every day.

	Depressed (n = 46)	Nondepressed (n = 189)	Odds Ratio (95% CI)	P. Value
Anhedonia	29 (63)	25 (13)	10.8 (5.2,22.6)	.001
Depressed Mood	27 (59)	18 (10)	12.4 (5.8, 26.4)	.001
Trouble Sleeping	32 (70)	17 (9)	22.7 (10.2, 50.6)	.001
Feeling Tired	32 (70)	28 (15)	12.9 (6.1, 27.2)	.001
Change in Appetite	30 (65)	17 (9)	18.6 (8.5, 40.8)	.001
Guilt/worthlessness	24 (52)	8 (4)	24.1 (9.7, 60.2)	.001
Trouble Concentration	26 (57)	6 (3)	40.8 (14.9, 111.6)	.001
Feeling slowed down or restless	20 (43)	10 (5)	13.3 (5.6, 31.7)	.001
Suicidality/thoughts of death	18 (39)	1 (.5)	131 (16.7, 1025.6)	.001

Sensitivity/specificity of the PHQ-2 to predict the PHQ-9 was assessed using the standard cut off of ≥ 3; sensitivity = 78.3, specificity = 85.6. A PHQ-2 cut off of ≥ 2 was calculated and revealed sensitivity = 91.3 and specificity = 71.6.

Discharge location did not vary between groups. Seventy four percent (34/46) of depressed patients went home, 11 (24%) to rehab; 143/187 (76%) non-depressed went home versus 44 (24%) to rehab.

The Mini-Cog was significantly related to educational level p = .026. 15/35 (43%) of those with <high school (HS) passed, 14/25 (56%) with HS passed, and 20/26 (77%) with college (plus) passed. However the Mini-Cog score was not linked with depression (p = .340).

## Discussion

Post stroke depression is important to identify and treat to optimize outcomes and recovery [1,3,6,7]. Early diagnosis on acute stroke units (within the first few days) has been shown to be feasible [4], and recent stroke guidelines now recommend early depression screening [17,18]. Although improvement of psychiatric assessment and subsequent mental health care are a high priority for the Middle East and North African (MENA) region [21], prevalence and intervention studies of post stroke depression are lacking.

Patients on the acute stroke ward at Hamad Hospital in Qatar were assessed for depression within the first few days post stroke. 19.7% scored in the moderately depressed range on the PHQ-9 (≥10 out of a possible 27). These results are consistent with the 18–30% depression prevalence rates found in studies conducted in Western countries within the first 2 weeks post stroke [3] and slightly higher that the 12 percent level of moderately depressed patients found within 4 days of admission [4]. Dysarthria was found to have a significant independent association with depression. Some prior studies have linked aphasia and dysarthria with higher rates of depression post stroke, though results have been variable [3,7].

Nationality was found to be a significant associated with depression. Middle Eastern and African (ME/A) patients had significantly greater depression rates (30.18%) than Southeast Asian and Western Pacific (SA/WP) (16.76%). This may be due to in part to different base rates of depression in the ME/A and SA/WP groups. The Middle East and African regions suffer from some of the world’s highest depression rates, while Southeast Asian and Western Pacific groups have some of the lowest [22,23]. Also, conflict in the Middle East may be a potential cause of increased depression, and citizens from many countries in the region have experienced trauma due to political instability. In addition, some studies have suggested that Arabic and African cultures have higher rates of stigma surrounding mental illness and the expression of psychiatric concerns, and this may diminish treatment seeking for depression [24,25].

The Minicog and NIHSS were interestingly not associated with post stroke depression in our sample. This is most likely related to the fact that strokes in Qatar tend to occur at a younger age and be less severe. The mean age was approximately 50 years in our sample and the mean NIHSS scores were approximately 4.5, a score in the minor range. The Minicog was also significantly related to educational level.

Item analysis of the PHQ-9 data revealed similar symptom profiles to those in stroke patients in the United States [26]. The most frequently endorsed items by depressed post stroke patients included; trouble sleeping, feeling tired, appetite changes and anhedonia (loss of interest or pleasure). In the non-depressed post stroke group, the most common symptom was feeling tired, however greater numbers endorsed anhedonia and depressed mood in Qatar than in the US (13 and 10% respectively versus 2 and 5%). Of note, endorsement of item 9 (suicide/feeling would be better off dead) was much higher in Qatar. 39% of our depressed post stroke patients felt they would be better off dead versus 10% in the US post stroke cohort [26]. Further study of suicidal ideation in post stroke patients in the Middle East is recommended given the high response rates found.

We assessed the PHQ-2 as a potential brief screener to simplify depression screening and increase the likelihood that screening will occur. The traditional cut off score for the PHQ-2 is ≥ 3, and this scoring is widely used in primary care settings [15]. Other studies have found that a cut off of ≥ 2 yields better sensitivity and specificity in stroke [10,16]. In our study a PHQ-2 cut off of ≥ 3 yielded sensitivity of 78.3 and specificity of 85.6 while a cut off score of ≥ 2 yielded a sensitivity of 91.3 and specificity of 71.6. Given the importance of not missing depression in the acute inpatient setting it is recommended that the cut off of ≥2 is also optimal for stroke patients in the MENA region.

Clinically we found that many post stroke patients were hesitant to take an antidepressant or receive follow-up care with psychiatry. A proactive, culturally sensitive approach to post stroke depression will likely be beneficial. Education is recommended for patients, their families and health care providers in order to reduce stigma regarding psychiatric treatment and care. Psychiatric programs embedded within medicine will likely improve treatment rates and outcomes. Prior literature has found that SSRIs are the first line treatment for post stroke depression, in conjunction with interventions that focus on care management, family support and psychoeducation [1,17].

Limitations of this study are that it did not include patients with more severe strokes who were unable to communicate effectively with the clinical staff due to confusion or aphasia. Research has shown that more severe strokes have higher depression rates, thus our data likely underestimates the total burden of depression. Socioeconomic factors were not available beyond education and nationality of origin. Variables such as economic status, living situation, etc. will be important to include in future studies. Another weakness in the study is that rates of depression pre-stroke were not obtained, as prior studies have identified depression as a risk factor for stroke, postulating that depressed patients may have worse self-care (e.g. exercising less, eating poorly) and also inflammatory markers may be elevated in both depression and stroke [27]. In addition, ideally all positive depression scores would have been verified with a gold standard such as the SCID (Structured Interview for the Diagnostic and Statistical Manual (DSM-4)) [28].

## Conclusion

This study supports the recent recommendation for acute post stroke depression screening, with screening found to be feasible on an acute stroke unit in Qatar. Moderate levels of depression (PHQ-9 ≥10) were found to be present in approximately twenty percent of patients. PHQ-2 screening is recommended as a quick and easier to administer first step, utilizing the lower cut score of ≥ 2 for stroke patients (versus the standard ≥ 3 for primary care patients), with patients obtaining positive screens being administered the full PHQ-9. It is important to consider nationality in depression assessment, as this study found Middle Eastern/African patients were more than twice as likely to present with depression than Southeast Asian/Western Pacific patients. Higher rates of thinking about death or suicide were noted making this an important area of further study. Education of patients and staff regarding depression as common co-occurring conditions post stroke is recommended in the MENA region, along with the provision of culturally sensitive psychiatric care.

## Supporting information

S1 Dataset Data acute post stroke depression middle east. (XLS)
