# Correction: Acute post stroke depression at a Primary Stroke Center in the Middle East

**DOI:** 10.1371/journal.pone.0212919

**Published:** 2019-02-21

**Authors:** Stacy Schantz Wilkins, Naveed Akhtar, Abdul Salam, Paula Bourke, Sujatha Joseph, Mark Santos, Ashfaq Shuaib

The following information is missing from the Funding section: This article was funded by the Qatar National Library. The funder had no role in study design, data collection and analysis, decision to publish, or preparation of the manuscript.
